# Involvement of Intestinal Goblet Cells and Changes in Sodium Glucose Transporters Expression: Possible Therapeutic Targets in Autistic BTBR T^+^Itpr3^tf^/J Mice

**DOI:** 10.3390/ijerph182111328

**Published:** 2021-10-28

**Authors:** Caterina Franco, Francesca Bonomini, Elisa Borsani, Stefania Castrezzati, Lorenzo Franceschetti, Rita Rezzani

**Affiliations:** 1 Anatomy and Physiopathology Division, Department of Clinical and Experimental Sciences, University of Brescia, 25123 Brescia, Italy; caterinafranco.1996@gmail.com (C.F.); francesca.bonomini@unibs.it (F.B.); elisa.borsani@unibs.it (E.B.); stefania.castrezzati@unibs.it (S.C.); lorenzofranceschetti@gmail.com (L.F.); 2 Interdipartimental University Center of Research “Adaption and Regeneration of Tissues and Organs-(ARTO)”, University of Brescia, 25123 Brescia, Italy; 3 Italian Society of Orofacial Pain (SISDO), 25123 Brescia, Italy

**Keywords:** autism spectrum disorder, BTBR mice, goblet cells, Sglt-1 and Sglt-3 proteins, light, ultrastructural and biochemical analyses

## Abstract

Autism spectrum disorder is a neurodevelopmental syndrome with a complicated etiology and could be responsible for disrupted gastrointestinal tract microbiota. The aim of this work was to study intestinal samples from an autistic animal model (BTBR mouse strain) to better describe gastrointestinal alterations. We performed a morphological and biological evaluation of small intestine samples. In terms of morphology, we studied the goblet cells, cells of intestinal mucosal responsible for the production and maintenance of the protective mucous blanket. Alterations in their secretion may indicate an altered rate of mucus synthesis and this is one of the possible causes of gastrointestinal problems. In terms of biological evaluation, impaired regulation of glucose homeostasis regulated by sodium-glucose transporters has been suggested as an important component of obesity and associated comorbidities; therefore, this study analyzed the expression of sodium/glucose transporter-1 and -3 in BTBR mice to better define their role. We demonstrated that, in BTBR mice as compared to C57BL/6J (B6) strain animals: (1) The goblet cells had different protein content in their vesicles and apparently a larger number of Golgi cisternae; (2) the expression and level of sodium/glucose transporters were higher. These findings could suggest new possible targets in autism spectrum disorder to maintain mucus barrier function.

## 1. Introduction

Autism spectrum disorder (ASD) is defined as a group of heterogenous neurodevelopmental conditions [1,2]. Recently, ASD diagnoses have been increased over a twenty-year period (1998–2018), reaching an overall 787% in its recorded incidence [3]. This disorder is becoming one of the most prevalent neurodevelopmental syndromes with a male–female ratio of 2.5:1 [4,5]. Moreover, ASD is a lifelong condition and associated with a very high number of comorbidities; these include gastrointestinal (GI) disturbance [6,7]; oral health problems [8]; and an increased risk of respiratory and sleep disorders and epilepsy [9,10,11].

Feeding problems are especially an issue for autistic children. They are very selective about types of food, texture and colour [12]; they choose energy-dense food rejecting fruits, vegetables and whole grains [6,13,14]. This leads to a greater probability of becoming overweight or even obese, conditions that can cause GI disorders, as proposed by Emerenziani et al. [15].

Obesity, GI disorders, and unbalanced gastrointestinal tract (GIT) are thought to be closely associated with ASD and could be responsible for disrupted GIT microbiota disorders [16].

While there are several animal models and human studies suggesting the link, as mentioned above, between alterations in GIT microbiota and the development of ASD, it is very difficult to reach definitive conclusions. More work is needed to outline the potential mechanisms involved in ASD in order to improve understanding of the complex morphological and cellular systems regulating the likelihood of developing this condition, as discussed by Rose et al. [17].

Taking together all the considerations about this disease and links to obesity and GIT alterations, the aim of this work was to study small intestinal samples from an autistic animal model (BTBR T+Itpr3tf/J or BTBR mice) to identify the markers involved in GIT alterations that are linked to being overweight. We used these animals since our starting point was the paper of Sotak et colleagues [18]. BTBR mice are considered a good ASD-like model that present behavioral and physiological alterations like those observed in patients with ASD. They are considered new models for ASD because initially they were mainly used for studies on insulin resistance, diabetes-induced nephropathy and phenylketonuria [19]. Only recently, they were characterized as having an autism-like behavioral phenotype [20,21]. They show a robust ASD phenotype being characterized by the three main symptoms: impaired sociability, communication deficits and repetitive behaviors [22,23]. Moreover, C57BL/6J (B6) mice models are available as the control strain for research using BTBR mice [24,25,26] and this has made it possible to use these models as the basis for many studies. Many of the studies carried out to date have focused on the psychological and behavioral component, but over the years research has become increasingly open, focusing on the metabolic, inflammatory, and global aspects of the autistic model [27,28,29]. In particular, the evidence, confirmed by several studies [19], showed that there is a real correspondence between the genetic alterations present in autistic subjects and those present in the animal models is fundamental. This made it possible to consider using this animal model for studies that did not focus solely on the psychological and behavioral components, extending the research to the organic and metabolic spheres [30]. According to these considerations, we performed an initial morphological and subsequent biological evaluation of small intestine samples.

In terms of morphology, we studied the goblet cells (GC), a type of intestinal mucosal cells responsible for the production and maintenance of the protective mucous blanket; these synthesize and secrete high-molecular-weight glycoproteins known as mucins. Other components within the mucous gel include water, electrolytes, sloughed epithelial cells and secreted immunoglobulins. Mucus produces a physical and chemical barrier that protects the epithelium from physical damage by luminal content, guards against bacterial invasion, regulates epithelial hydration, and interacts with secreted immunoglobulin A to produce antibody and antitoxin effects [23,31,32]. Recently, Yang and Yu [33] indicated that the perspective regarding GCs and their products has changed, suggesting that they are not passive cells but play a positive role in maintaining intestinal tract immunity and mucosal homeostasis. They can obtain luminal antigens, presenting them to the antigen presenting cells that induce adaptive immune response. Alterations in GC secretion may indicate an altered rate of mucus synthesis and may be evidence of one of the possible causes of GIT problems [34]. Moreover, the changes in the mucus index are markers of several intestinal diseases, such as infection, inflammatory bowel disease and cancer [35,36]. The new role of GCs in immune surveillance [37] has opened the possibility suggesting that GC could be a new target in the treatment of several inflammatory diseases and food allergy.

In terms of biological evaluation, it is well-known that intestinal absorption of nutrients is more rapid and efficient in obese than in lean humans [38]; impaired regulation of glucose homeostasis regulated by sodium-glucose transporters, also known as sodium/glucose cotransporters (Sglt-1 and Sglt-3), has been suggested as an important component of obesity and associated comorbidities. The recent evaluation of a homolog of Sglt-1, Sglt-3, found its expression changed in this pathological condition [18]. Thus, the other aim of this study was to characterize the expression of Sglt-1 and Sglt-3 in BTBR mice to highlight the important role of glucose transporters in ASD.

Through these investigations, we hope to improve knowledge of GIT morphology and its susceptibility to diseases. Additionally, this study could help to improve our understanding of the pathogenesis of ASD and identify more specific targets for therapies and even tailored interventions.

These strategies could aim to modify the composition of GIT microbiota to make the intestinal epithelial barrier more effective and reverse the GIT alterations to reduce issues of excess weight and other comorbidities found in autistic children.

## 2. Materials and Methods

### 2.1. Experimental Groups

Twenty male BTBR T+Itpr3tf/J (JAX™ Mice Strain) mice—as transgenic animal model of ASD—and twenty C57BL/6J (B6) (JAX™ Mice Strain) mice—as control (CTR) strain mice—starting from 3 weeks of life were housed in cages (2 or 3 animal/cage), with food and water ad libitum and kept in an animal house at a constant temperature of 20 °C, with 12 h alternating light–dark cycle to minimize the circadian variations.

The body weight was monitored and evaluated during the 13 weeks for each animal.

Before the beginning of the experiment, mice were left housed in the animal facility for 1 week. All efforts were made to minimize animal suffering and the number of animals used. All the experimental procedures were approved by the Italian Ministry of Health and followed the National Institutes of Health guide for the care and use of Laboratory animals (NIH Publications No. 8023, revised 1978).

Each mouse at the age of 13 weeks was deeply anesthetized (isoflurane 5%) and transcardially perfused with sterile saline (0.9% NaCl) according to Stacchiotti and colleagues [39]. Then, the animals were, again, perfused by 1 L of 4% paraformaldehyde in phosphate buffer saline (0.1 M, pH 7.4). For morphological, immunohistochemical and ultrastructural evaluation, the gut was carefully removed from each mouse.

### 2.2. Sample Processing

The small intestine samples were rinsed in sterile saline and each sample was divided in two different portions. One—used for the morphological and histochemical evaluations—was dehydrated in graded ethanol, and then embedded in paraffin wax according to standard procedures. The other part was fixed in 2.5% glutaraldehyde in cacodylate buffer 0.1 M (pH 7.4) for 3 h at +4 °C and postfixed in 2% osmium tetroxide in cacodylate buffer for 1 h at +4 °C for the ultrastructural investigation.

### 2.3. GIT Morpho-Histological Assessment

Serial paraffin sections (5 μm thick) of each sample were cut with a microtome [40]. Alternate paraffin sections were deparaffinized, rehydrated, and stained with hematoxylin and eosin (Bio Optica, Milan, Italy) and then were observed with an optical light microscope (Olympus, Hamburg, Germany) at a final magnification of 20×.

Digital images of the intestinal mucosa, villi, criptae and GCs were captured using a light microscope (Olympus, Hamburg, Germany).

### 2.4. Goblet Cell Evaluations

For histochemical analysis, next to hematoxylin and eosin staining, specimens from small intestine were collected and stained with (a) periodic acid-Schiff (PAS)-Alcian Blue—this procedure was performed to distinguish neutral and acidic and mixed mucins (stained in red, blue, and purple, respectively), in GCs; (b) Xylidine-Ponceau, for better describing the total protein content of the GCs.

For the first staining, two slides containing 3–4 sections were prepared from each sample. Next, sections (5 µm) were deparaffinized with xylene and dehydrated in ethanol solutions. Tissues were oxidized in 0.5% periodic acid solution for 5 min and rinsed in distilled water. Subsequently, sections were stained in Schiff reagent for 10 min and rinsed in tap water for 5 min. After that, sections were stained with 1% Alcian Blue solution in 3% aqueous acetic acid (pH 2.5) for 15 min. In the end they were counter-stained with Hematoxylin (Carazzi’s Emallumen) [34].

Regarding the Xylidine-Ponceau staining, this histochemical technique was performed according to the protocol [34] to show the protein content in the cells, as well as in the secretion of GC.

At the end, all the samples were observed with an optical light microscope (Olympus, Hamburg, Germany) at a final magnification of 100×.

### 2.5. Immunolocalization of Sglt-1 and Sglt-3

Briefly, the sections were subjected to antigen retrieval in 0.05 M sodium citrate buffer (pH 6.0) in hot water bath (98 °C for 20′) and then incubated firstly in adequate serum (10% in TBS plus 0.1% Triton X-100) for 60 min and then in primary antiserum directed against: Sglt-1 (anti-Sglt-1, rabbit polyclonal antibody, Merck KGaA, Darmstadt, Germany, diluted 1:100) and Sglt-3 (anti-Sglt-3, rabbit polyclonal antibody, Proteintech Group, Inc., Manchester, UK, diluted 1:100).

After incubation in the primary antiserum, the sections were sequentially incubated with appropriated biotinylated secondary antibodies and avidin-biotin peroxidase complex (Vector Labs., Burlingame, CA, USA). The reaction product was visualized using hydrogen peroxide and diaminobenzidine (Sigma, St. Louis, MO, USA) as chromogen; the immunopositivity was identified as a brown colour. To better visualize the positive reaction, the sections were counterstained with Carazzi’s Emallumen (blue/violet colour), dehydrated, and mounted with DPX, for light microscopy detection.

The immunohistochemical control was performed by omitting the primary antibody and incubating the sections with non-immune rabbit serum and with isotype-matched irrelevant rat IgGs as negative control. All the samples were observed with an optical light microscope (Olympus, Hamburg, Germany) at a final magnification of 10×.

The immunopositivity was evaluated quantitatively by an optical microscope (Olympus, Hamburg, Germany).

At the end, all the samples were observed with an optical light microscope (Olympus, Hamburg, Germany) at a final magnification of 100×.

Digitally fixed images were analyzed using an image analyzer (Image Pro-Plus, Milan, Italy) by researchers unaware of the health condition of the mice and were calculated as percentage of positive area (%) according to Borsani and colleagues [41,42]. The analysis has been performed on five sections for each sample evaluating, for each section, six random fields with the same area (52 × 103 μm^2^).

### 2.6. Transmission Electron Microscopy

Three small intestinal samples obtained from GIT of each mouse (10 animals for BTBR and 10 mice for CTR) were fixed in glutaraldehyde and were treated for ultrastructural analysis according to Rezzani et al. [40]. Briefly, adipose tissues were dehydrated in increasing ethanol concentrations and propylene oxide, followed by Araldite-Epon resin embedding. Semithin sections (1 μm thick) were obtained using an UltraCut E ultramicrotome, then stained by toluidine blue and observed with a light microscope (Olympus, Hamburg, Germany). Subsequently, from representative blocks, 70 to 80 nm-thick ultrathin sections were obtained using a diamond knife, collected on formvar-coated grids, double stained by uranyl acetate and lead citrate, and observed with a transmission electron microscopy (Tecnai G2 Spirit; FEI Company, Eindhoven, the Netherlands) at 80 kV.

Two blinded observers evaluated 10 images for each sample (3 samples) for each animal (10 animals for BTBR and 10 mice for CTR); in these images, nucleus and vesicles of GCs were always evident and the same have been performed in perinuclear area where the Golgi apparatus is normally present [43]. The observers carried out a semiquantitative analysis indicating from 4 to 8 cisternae as normal presence of the latter (+) and with several cisternae higher than 8 greater presences of cisternae (++). The evaluation of physiologically cisternae presence has been made according to Jelerčič U. [44].

The blinded investigators performed the analysis, and their evaluation was assumed correct if their values were not significantly different. If there was disagreement concerning the interpretation, the case was reconsidered in order to reach a unanimous agreement.

### 2.7. Western Blot Sglt-1 and Sglt-3 Evaluation

According to Vanella and colleagues, the small intestine homogenates of each experimental groups homogenized with the aid of a Polytron homogenizer (IKA Works Inc., Wilmington, NC, USA) in ice-cold Tris- buffered saline containing a protease-inhibitor cocktail, and after mixing with sample loading buffer (50 mM Tris-HCl, 10% *wt*/*vol* sodium dodecyl sulfate, 10% *vol*/*vol* glycerol, 10% *vol*/*vol* 2-mercaptoethanol, and 0.04% bromophenol blue) in the ratio of 4:1, they were boiled for 5 min [45]. In detail, samples (30 mg proteins) were loaded onto 8% or 12% sodium dodecyl sulfate polyacrylamide gel electrophoresis gels and subjected to electrophoresis (120 V, 90 min). The separated proteins were transferred to nitrocellulose membranes (Bio-Rad, Hercules, CA, USA). After transfer, the blots were incubated with LI-COR blocking buffer (LI-COR Biosciences, Lincoln, NE, USA) for 1 h, followed by overnight incubation with a 1:1000 dilution of the primary antibody. Primary polyclonal antibodies directed against Sglt-1, Sglt-3 and β-Actin (rabbit polyclonal antibody anti-Sglt-1, Merck KGaA, Darmstadt, Germany; rabbit polyclonal antibody anti-Sglt-3, Proteintech Group, Inc., Manchester, UK; rabbit polyclonal antibody anti β-Actin GTX109639, GeneTex, Irvine, CA, USA).

After washing with Tris-buffered saline, the blots were incubated for 1 h with the secondary antibody (1:1000). Protein detection was carried out using a secondary infrared fluorescent dye conjugated antibody, absorbing at 800 and 700 nm. The blots were visualized using an Odyssey Infrared Imaging Scanner (Li-Cor Inc., Lincoln, NE, USA) and quantified by densitometric analysis performed after normalization with β-Actin using a computer-assisted densitometer (Rasband, W.S., ImageJ, U.S. National Institutes of Health, Bethesda, Maryland, MD, USA).

### 2.8. Statistical Analysis

Results are expressed as mean ± standard deviation (SD). Statistical significance of differences among the experimental groups for all the markers was evaluated by analysis of variance (one way ANOVA calculated by Origin^®^ 7SRI, 1991–2002 OriginLab Corporation, One Roundhouse Plaza, Northampton, MA 01060 USA) corrected by Bonferroni test with significance set at *p* ≤ 0.01 for the immunohistochemical analysis and at p ≤ 0.05 for GC quantification. The results of quantitative immunohistochemical evaluation were compared between both healthy mice and ASD-model mice at the same age.

Groups were compared by analysis of variance (ANOVA) followed by the Bonferroni correction for multiple comparisons (significant at *p* < 0.05).

## 3. Results

BTBR and CTR mice remained healthy during the whole experiment, consuming readily their daily food. During the 13 weeks the weight of the mice was monitored, and it was found that there was a significative difference between the CTR and ASD group, as reported in Table 1. In general, we have found that autistic models still maintain a higher body weight during the growth, showing an increase in body weight which, throughout the 13 weeks, remains significant (*p* < 0.0001). This is only a baseline evaluation, but it could be considered a starting point for other in-depth analysis in order to identify a possible correspondence between a clinical variation—body weight—and morphology and immunohistochemistry patterns that characterize both the morphology of the villi and the content and number of GCs in ASD models.

### 3.1. Light Microscopy

#### 3.1.1. Hematoxylin and Eosin

This histochemical technique was used for demonstrating the general cytoarchitecture of the GIT samples (i.e., mucosa and submucosa).

**Figure 1:**
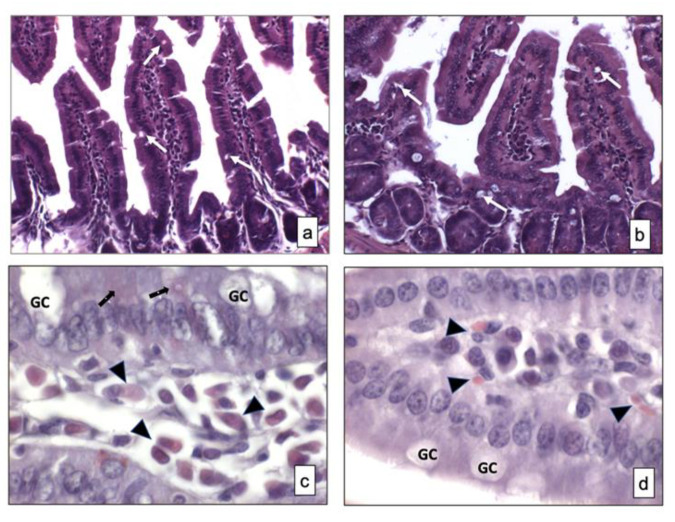
Overview of small intestine samples in BTBR (**a**,**c**) and CTR (**b**,**d**) mice respectively stained using hematoxylin and eosin and Xylidine-Ponceau techniques. Goblet cells are indicated as white arrows in (**a**,**b**) and as GC in (**c**,**d**); Lymphatic cells (i.e., plasma cells) are indicated with arrowheads in (**c**,**d**); Epithelial cells are indicated as black arrows in (**a**,**b**). (**a**,**b**)—200×; (**c**,**d**)—1000×. The number of animals was 10 for BTBR and 10 for CTR; moreover, three samples for each animal were used. The images are a representative sample of all the tissues examined.

No differences in the morphology of the small intestine samples from two strains of mice were observed (Figure 1a,b). Signs of relevant alterations such as cellular vacuolization and other types of changes were not observed in BTBR mice compared to CTR animals. Moreover, the GCs were not stained in both strains of mice, as visible in Figure 1a,b.

#### 3.1.2. Xylidine-Ponceau

This histochemical technique was used for identifying the protein content in the mucosa and submucosa of two strains of mice.

Weak positive cells staining was observed in the small intestine mucosa of both strains of mice and some positive cells were found in the submucosa of the same animals. There were apparently more cells in the submucosa of BTBR mice, and, based on their morphology, they could be classified as lymphatic cells (i.e., plasma cells). In fact, these cells were found in the cheliferous vessels of the submucosa (Figure 1c,d); the mucus secreted by the GIT-GCs was not stained by this technique (Figure 1c,d).

#### 3.1.3. Periodic Acid-Schiff-Alcian Blue

This technique was used for the specific identification of acid, neutral and mixed mucins in the GCs of BTBR and CTR mice.

**Figure 2:**
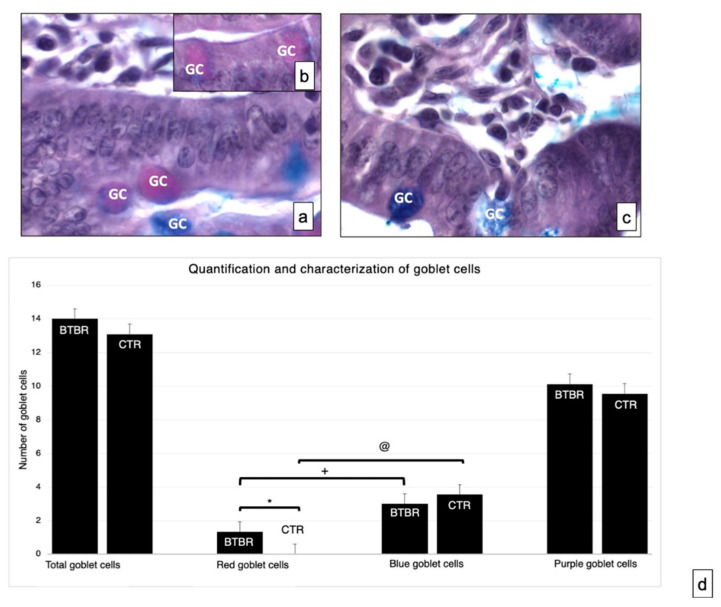
Representative histological images stained using PAS-Alcian Blue technique, which allows the identification of acid (blue) and neutral (red) and mixed (purple) carbohydrates. Goblet cells—GC. (**a**,**b**)—BTBR mice; (**c**)—CTR animals. (**a**–**c**)—1000×; (**d**)—quantification and characterization of goblet cells. Data are presented as mean ± SD. * *p* < 0.05 Red goblet cells, BTBR mice vs. CTR mice; + *p* < 0.05 red goblet cells vs. blue goblet cells in BTBR mice; @ *p* < 0.05 red goblet cells vs. blue goblet cells in CTR mice. The number of animals was 10 for BTBR and 10 for CTR; moreover, three samples for each animal were used. The images are a representative sample of all the tissues examined.

Neutral and acid mucosal substances were observed in the GCs in the mucosa of experimental groups (Figure 2a–c). We assessed the total number of GCs in BTBR and CTR mice. The total number of GC in BTBR mice was higher than that observed in CTR animals, although it was not statistically significant (Figure 2d).

As reported in Materials and Methods, the GC showed acid mucins stained in blue, neutral mucins in red and mixed mucins in purple. The number of cells with neutral mucins in BTBR mice was very low; instead, the number of the same cells positive for acidic mucins was high, as was the number of purple cells (Figure 2b).

**Figure 3:**
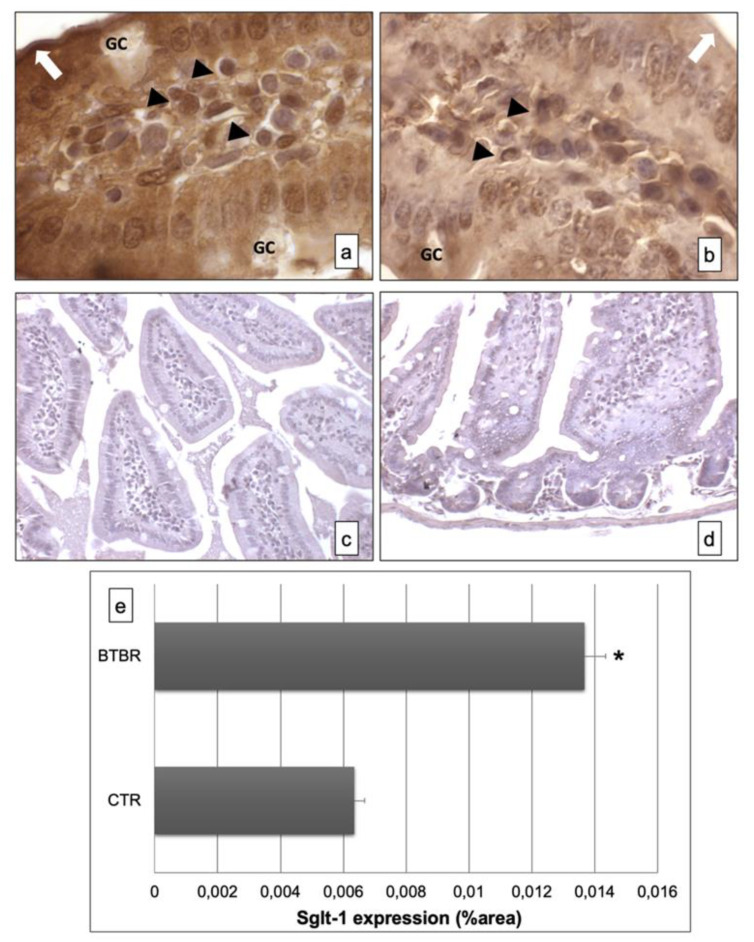
Representative images of Sglt-1 immunostaining in small samples of BTBR and CTR mice ((**a**,**b**) respectively). The immunohistochemical signal (brown) is localized in the intracellular compartment and apical portion (highlighted by a white arrow) of small intestine and in some lymphatic cells of the submucosa. The negative controls of immunohistochemistry are shown in images (**c**,**d**) for BTBR and CTR animals respectively. (**e**) Statistical analyses of this immunohistochemical technique for intracellular compartment and apical portion of small intestine. Data are presented as mean ± SD. * *p* < 0.01 vs. CTR mice. Goblet cells are indicated as GC; lymphatic cells (i.e., plasma cells) are indicated with arrowheads (**a**,**b**); apical portion of the enterocytes are indicated as white arrows in (**a**,**b**)—1000×. The number of animals was 10 for BTBR and 10 for CTR; moreover, three samples for each animal were used. The images are a representative sample of all the tissues examined.

We compared the number of acid cells (blue) and neutral cells (red) in both CTR and BTBR. We have seen that in both cases the number of acid (blue) cells is statistically higher than the number of neutral (red) cells (in Figure 2d the significance for BTBR red cells vs. blue cells is indicated with +; the significance for CTR red cells vs. blue cells is indicated with @). Data that have to be considered concern the comparison between BTBR and CTR. In this case the difference was significant only considering the number of neutral (red) cells, showing that these are significantly higher in the BTBR (in Figure 3d the significance is indicated with *).

### 3.2. Immunohistochemical Analysis of Sglt-1 and Sglt-3

To evaluate the presence of Sglt-1 and Sglt-3, we assessed their expressions by an immunohistochemical study using Sglt-1- and Sglt-3-specific polyclonal antibodies on small intestine sections from two observed strains of mice.

The immunohistochemistry for Sglt-1 showed, in BTBR mice, clear and strong positivity in the intracellular compartment and apical portion of the enterocyte lining in the mucosa of intestinal villi (Figure 3a). Instead, the positivity was moderate and lower in CTR mice (Figure 3b). The GCs were negative in both the strains of mice.

The negative controls of immunohistochemistry were similar in both strains of mice, as shown in Figure 3c,d.

Moreover, we also found some cells positive for this protein in the submucosa of BTBR and CTR mice; these cells were higher in BTBR animals compared to CTR mice and they could be plasma cells. Instead, the GCs were negative (Figure 3a,b).

All results were similar to those observed by quantitative evaluations as reported in Figure 3e.

**Figure 4:**
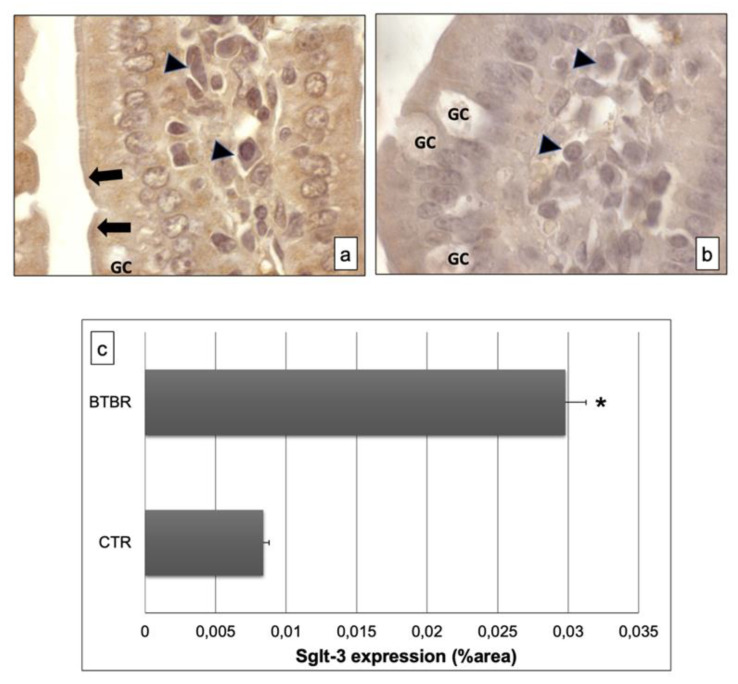
Representative images of Sglt-3 immunostaining in small samples of BTBR and CTR mice ((**a**,**b**) respectively). The immunohistochemical signal (brown) is localized in the intracellular compartment and apical portion (highlighted by black arrows) of small intestine and in some lymphatic cells of the submucosa. (**c**) Statistical analyses of this immunohistochemical technique for intracellular compartment and apical portion of small intestine. Data are presented as mean ± SD. * *p* < 0.01 vs. CTR mice. Goblet cells are indicated as GC; lymphatic cells (i.e., plasma cells) are indicated with arrowheads in (**a**,**b**); brush border membrane is indicated as black arrows in (**a**,**b**).

The immunohistochemistry of Sglt-3 revealed moderate positivity in the intracellular enterocytes and in the epithelial brush border membrane of BTBR mice samples; we also observed some cells in the submucosa positive for this antibody (Figure 4a). Notably, the positivity was weak in intracellular enterocytes and also in the brush border membrane in CTR mice. Furthermore, the lymphatic cells, which could be plasma cells, in the submucosa showed moderate staining for this antibody in BTBR mice and weak positivity in CTR animals (Figure 4b).

These results were also confirmed by immunomorphometrical analyses (Figure 4c).

The specificity of this antibody was observed in the same way as for Anti-Sglt-1 (data not shown).

### 3.3. Transmission Electron Microscopy

**Figure 5:**
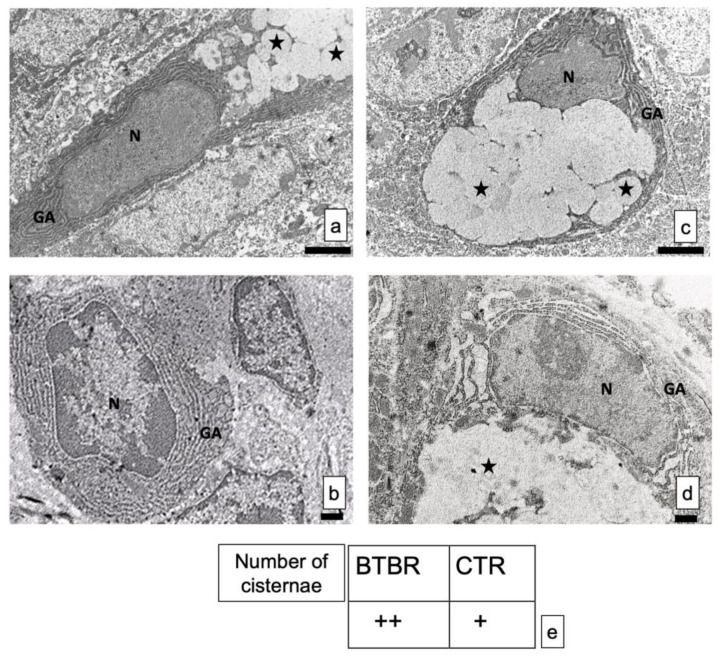
(**a**–**d**) Representative ultrastructural images of intestinal goblet cells in BTBR and CTR mice. (**a**,**b**) BTBR mice; (**c**,**d**) CTR animals. Vesicles of goblet cells are indicated as black stars; nucleus of goblet cells is indicated as N; Golgi’s apparatus is indicated as GA. Bar: (**a**,**b**)—2 μm; (**c**,**d**)—1 μm. (**a**–**c**)—6200×; (**b**,**d**)—8700×; (**e**) semiquantitative evaluation of cisternae in Golgi apparatus of BTBR and CTR animals. The number of animals was 10 for BTBR and 10 for CTR; moreover, three samples for each animal were used. The images are a representative sample of all the tissues examined. (+): from 4 to 8 cisternae and (++): several cisternae higher than 8.

The GCs did not show alterations in shape and size, in all experimental groups. The nuclei were very clear with electrodense heterochromatin (Figure 5a–d).

The structure of the cisternae in the Golgi apparatus did not show perceptible differences between BTBR and CTR mice. There were no alterations in their shape or size, although apparent changes in number of cisternae in the cells can be seen. In fact, BTBR mice, when compared to CTR mice, showed apparently a higher number of Golgi apparatus cisternae in these cells (Figure 5c). However, CTR mice had the same pattern of distribution in all GCs observed in these animals. Figure 5d showed a representative GC with its nucleus, Golgi apparatus and vesicles.

These results were confirmed by semiquantitative analysis (Figure 5e).

### 3.4. Western Blot Analyses

**Figure 6:**
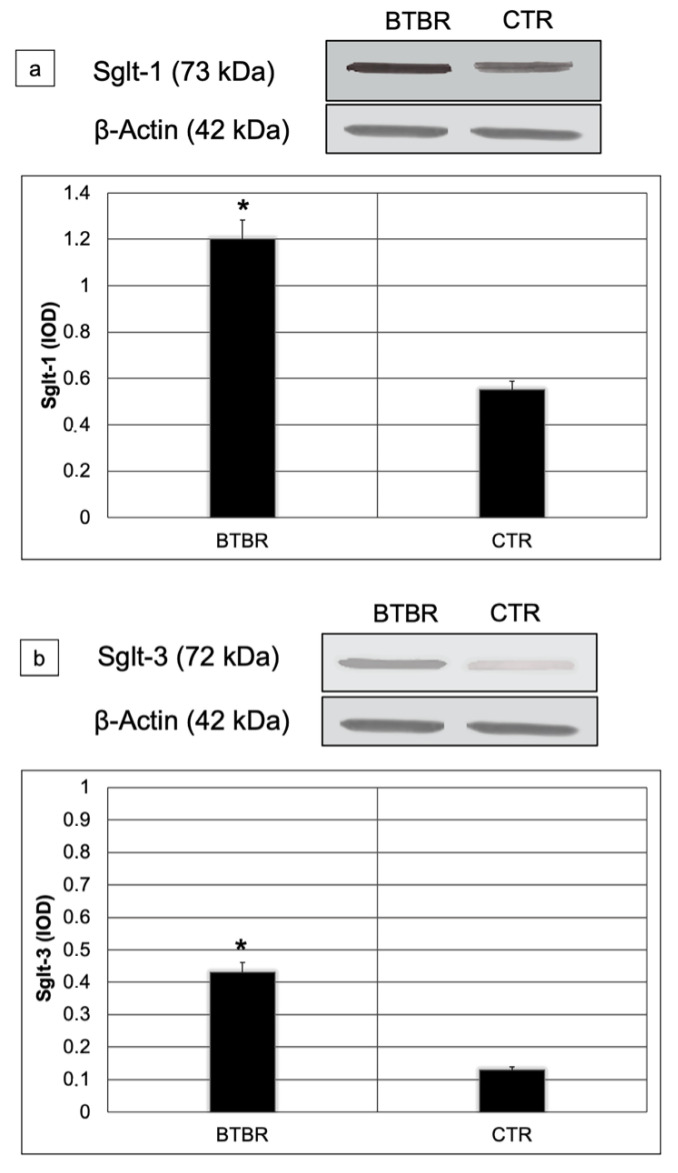
Representative blot and densitometric analysis of Sglt-1 (**a**) and Sglt-3 (**b**) proteins levels in the small intestine of studied groups. Data are presented as mean ± SD. * *p* < 0.01 vs. CTR mice.

We assessed Sglt-1 and Sglt-3 protein levels using Western blot in small intestine lysates from all studied mice. Sglt-1and Sglt-3 abundances were significantly higher in BTBR compared to CTR mice (respectively 1.2 vs. 0.6; *p* < 0.01 for Sglt-1 and 0.42 vs. 0.12 *p* < 0.01 for Sglt-3) (Figure 6a,b).

## 4. Discussion

To our knowledge, this is the first study to evaluate the GC content in autistic animals, although they have been found to play a role in the development of neurological disorders [35]. Our findings demonstrated that the secretion of GCs changed in BTBR mice compared to CTR animals; there were more neutral (red) mucins than acidic (blue) mucins as visualized by light microscopy. Furthermore, these results were confirmed by EM evaluations in which we demonstrated increased activity of these cells linked to Golgi’s apparatus. As reported in the Introduction, GCs are specialized cells with specific mechanisms for the secretion of mucus, a complex glycoprotein gel that covers the surface of epithelium villus and contributes significantly to cell protection, giving several helps to the microbiota [34]. GCs are found the entire length of the GIT [46]. The mucus is a complex aqueous fluid that owes its properties to glycoprotein mucin combined with electrolytes, lipid and other proteins [47]. Moreover, mucus is a natural and biological selective habitat for the gut microbiota since it serves as attachment sites for bacteria, promoting their colonization [48]. The changes of mucus composition allow commensal and pathogenic microorganisms to reach the intestinal epithelium inducing infections and inflammation, as reported in many diseases [49]. So, our results, demonstrating changes in mucus secretion with a predominance of acidic or mixed secretion (blue or purple staining), led us to suggest and emphasize the “leaky gut” hypothesis as reported by Oh et al. [50]. This hypothesis is connected to the fact that a variation in intestinal permeability associated to GCs can cause changes in the signals of intestinal permeability and, consequently, lead to signals that are not physiologically correct for the body’s metabolism. Furthermore, Herath et al. [46] reported that changes in mucus properties can alter commensal microbial populations and cause dysbiosis; this alteration has been observed in patients with neurological diseases and it can contribute to the progression of the disorder by modifying gut barrier function (i.e., by altering the thickness of the mucus). These data are compatible with a thinner mucus layer as reported by Paone et al. [48]. These Authors demonstrated that changes in the microbiota community are also linked to aging and they suggested a possible relationship with the well-known changes in adiposity and the regulation of energy homeostasis [51]. Thus, for this point we suggest that there is a close relationship between GCs and microbiota, since the cells produce mucus for maintaining the composition of the gut microbiome [32]; therefore, mucus secretion and mucus layer formation protect the intestinal mucosal barrier [52].

The ultrastructure analyses showed apparently larger quantities of Golgi apparatus in BTBR mice demonstrated by semiquantitative analysis. These results could suggest a higher rate of synthesis of secretory vesicles. Thus, these results may strengthen the idea that an increase in intestinal secretion could be one of the possible causes of diarrhea among autistic, obese, and diabetic patients [53]. Moreover, in agreement with Gartner and Hiat’s data [54], we propose that the increase in rate of synthesis of secretory vesicles could mean the GIT is debilitated by these conditions.

For better explaining and focusing our findings, we carried out biological and molecular studies of two sodium/glucose cotransporters. We studied the Sglt-1 protein that is responsible for transporting the monosaccharide from the intestinal lumen to enterocytes [55] and Sglt-3, which is considered a glucose sensor [18]. In agreement with the role of these proteins, their expressions were different in BTBR and CTR mice. Their expressions and levels were upregulated and downregulated in BTBR and CTR mice, respectively. These results could be associated with the acceleration of glucose absorption and glucose sensor proteins, linking, or following pathophysiological states, such as hyperglycemia after fasting [55].

In this regard, however, it is important to underline the difference in results compared to those obtained by Sotak et al. [18]; in fact, these authors showed that diabetic BTBR ob/ob mice showed downregulation of Sglt-3 and not upregulation as we have demonstrated. The different results can be explained because it is true that we used the same strain of animals, but in the case of Sotak et al. [18] the animals were diabetic and obese. Instead, our results showed no difference between the groups in relation to body weight.

Because the increased expression of Sglt-1 has been shown to be associated with glucose absorption [56], these observations indicate that abnormal glucose absorption may play an important role in ASD. In our opinion, these results can be linked to Sglt-1 protein but also to glucose sensors, such as Sglt-3 protein. This protein plays a role in coordinating intestinal functions and it can reduce glucose absorption [57,58].

## 5. Conclusions

In this study, we demonstrated that, in BTBR mice, there is an alteration in the normal pattern of presentation of GCs. Few researches exist on this subject, particularly in mice models. Our results, however, agree with the findings of James et al. [59], who demonstrated that, in adult *shank3ab*ΔC^−/−^ animals, GCs were significantly increased as compared to wild type. We, therefore, believe that these data could be a starting point for a more in-depth evaluation of the role of GCs in the pathogenesis of ASD.

Secondly, we also demonstrated that, in BTBR mice compared to CTR animals: (1) Autistic models still maintain a higher body weight during the growth, showing an increase in body weight which, throughout the 13 weeks, remains significant; (2) the GCs had different protein content and apparently a higher number of Golgi cisternae; (3) the expressions and levels of Sglt-1 and Sglt-3 were very higher. Moreover, no difference of body weight was evident between the groups.

In conclusion, these data could be considered a starting point for focusing on the role of GCs and the intestinal expression of Sglt-1 and Sglt-3. These findings could suggest possible new targets in autism spectrum disorder for maintaining mucus barrier function.

Moreover, since there are many doubts about the links between microbiota alterations and ASD, our results could be considered a further study for better evaluating the relationship between them, even though we are aware that it is not known whether changes to microbiota are causal factor of ASD or if it is the disorder that causes the microbial alterations. Thus, further research is essential to evaluating and identifying the markers of the etiologies and pathological mechanisms of ASD.

## Figures and Tables

**Table 1:** Overview of body weight (g) evaluated weekly during the lifespan in the experimental animals: BTBR and CTR mice. Table represents detailed data about weight expressed as mean ± SD, * *p* < 0.0001 BTBR mice vs. CTR obtained by one-way ANOVA and followed by the Bonferroni correction for multiple comparisons (significant at *p* < 0.05).

	BTBR		CTR	
Weeks of Life	Weight Mean (g)	SD	Weight Mean (g)	SD
3	19.6 *	1.41	10.45	0.95
6	24.98 *	1.26	15.9	1.76
7	28.9 *	1.53	18.29	1.84
8	31.2 *	2.10	21.05	1.83
9	32.15 *	2.38	23.41	1.61
10	31.64 *	2.82	24.55	2.02
11	32.97 *	2.35	25.44	1.31
12	33.86 *	2.18	26.25	1.00
13	34.29 *	2.30	27.19	1.46

## Data Availability

Not applicable.
